# The Mediating Role of Active Coping Strategies in the Relationship Between Academic Stressors and Stress Responses Among University Students

**DOI:** 10.3390/healthcare13141674

**Published:** 2025-07-11

**Authors:** Cristina Ruiz-Camacho, Margarita Gozalo, Inmaculada Sánchez Casado

**Affiliations:** 1 Department of Psychology and Anthropology, Faculty of Education and Psychology, University of Extremadura, 06071 Badajoz, Spain; cristinarc@unex.es (C.R.-C.); iscasado@unex.es (I.S.C.); 2 Department of Psychology and Anthropology, Faculty of Sport Science (Psychology Laboratory), University of Extremadura, 10005 Caceres, Spain

**Keywords:** academic stress, stress responses, active coping strategies, university students, multiple mediation

## Abstract

**Background/Objectives**: Academic stress is a major factor affecting university students’ psychological well-being and overall functioning. This study examined whether three active coping strategies—positive reappraisal, social support seeking, and strategic planning—mediate the relationship between academic stressors and self-reported stress responses. **Methods**: A quantitative, cross-sectional, non-experimental design was employed. The sample comprised 1014 students from the University of Extremadura (M*_age_* = 20.56, SD = 3.50). Three subscales of the Academic Stress Questionnaire (CEA) were administered: Academic Stressors (E-CEA), Stress Responses (R-CEA), and Coping Strategies (A-CEA). Descriptive statistics, correlation analyses, and a multiple mediation model using structural equation modeling (SEM) tested direct and indirect effects, controlling for gender, study year, and academic field. **Results**: (1) Academic stressors were inversely related to positive reappraisal (*β* = −0.34, *p* < 0.001), planning (*β* = −0.12, *p* < 0.001), and social support seeking (*β* = −0.09, *p* < 0.01). (2) All three coping strategies were significantly associated with fewer stress symptoms, with positive reappraisal showing the strongest effect (*β* = −0.13, *p* < 0.001), followed by social support seeking (*β* = −0.06, *p* < 0.05) and planning (*β* = −0.03, *p* < 0.05). (3) Stressors had a strong positive direct effect on stress responses (*β* = 0.54, *p* < 0.001). (4) Coping strategies partially mediated the stressor–symptom link (total indirect effect: *β* = 0.12, *p* < 0.001, 95% CI [0.08, 0.16]). **Conclusions**: Active coping partially buffers the negative effects of academic stressors on perceived distress. Findings underscore the importance of enhancing students’ coping skills and implementing institutional policies that reduce structural stress and support psychological well-being.

## 1. Introduction

The university context represents a critical stage in student development, marked by life transitions, new responsibilities, and heightened academic demands [1,2]. A growing body of evidence has identified university students as particularly vulnerable to stress—a condition that not only undermines immediate well-being but also acts as a risk factor for more severe disorders such as anxiety and depression [3,4,5]. Comparative studies suggest that higher education students report greater levels of stress than their non-university peers [6], and that recent cohorts appear to face increased difficulty in managing academic pressure compared to previous generations [7,8].

Recent research indicates that a significant proportion of university students experience elevated levels of academic stress, stemming from factors such as academic overload, evaluative pressure, decreased motivation, and difficulties in emotional regulation [9,10,11]. This burden is often exacerbated in specific student populations facing additional challenges—such as social isolation, financial insecurity, or cultural adjustment—combined with limited coping resources [12,13]. Further compounding the issue are institutional gaps in support systems and a pervasive reluctance to seek professional help, frequently driven by stigma-related concerns.

In line with these findings, institutional reports—such as that issued by the University of Extremadura [14]—highlight the widespread prevalence of anxiety, coping difficulties, and increased risk of social maladjustment, emphasizing the need for systemic interventions responsive to the evolving demands of the educational environment. Prolonged exposure to stress has also been associated with the emergence of maladaptive behaviors, including substance use [15,16]. Moreover, chronic stress has been shown to impair core cognitive functions such as attention and memory [17,18], thereby undermining academic performance and increasing the likelihood of university dropout [19,20,21].

A comprehensive understanding of academic stress requires moving beyond reductionist approaches that focus solely on observable symptoms and instead considering the psychological and relational processes that mediate between environmental demands and individual responses. This conceptual shift has led to the development of theoretical models structured around three main levels of analysis: the individual, the contextual, and their dynamic interaction. Chust-Hernández et al. [22] underscore the contemporary relevance of the person–environment approach, as it offers a more nuanced and contextually grounded interpretation of academic stress.

Within this framework, the systemic-cognitivist model [23,24]—grounded in general systems theory [25] and the transactional model of stress [26]—conceptualizes the individual as an open system engaged in continuous interaction with the environment. The system’s equilibrium is disrupted when external demands (input) exceed the student’s internal resources, resulting in an imbalance expressed through stress responses defined as a constellation of physiological, psychological, and behavioral changes mobilized by the organism to cope with perceived threats or challenges [9,16]. This disruption activates self-regulatory mechanisms (output), primarily coping strategies, aimed at restoring homeostasis.

Lazarus and Folkman [26] (p. 141) define coping as “constantly changing cognitive and behavioral efforts to manage specific demands that are appraised as exceeding or taxing the resources of the individual.” This process involves two key components: a primary appraisal, in which the nature and severity of the threat are evaluated, and a secondary appraisal, in which available resources to address it are assessed. Coping thus operates as a mediating mechanism between the stressor and its outcomes, modulating the intensity of the emotional response and shaping the individual’s adaptive capacity [27].

Coping strategies vary according to the perceived level of control and include both problem-focused and emotion-focused approaches. These range from active, approach-oriented strategies—such as planning and positive reappraisal—to avoidant responses like denial and emotional disengagement [28,29]. Crucially, these strategies are not mutually exclusive; rather, individuals often combine them flexibly, depending on the characteristics of the stressor and the surrounding contextual demands [30,31,32].

Within the university context, research has consistently identified three active coping strategies as particularly relevant for their positive impact on students’ adaptation to academic demands: positive reappraisal, social support seeking, and strategic planning [33]. These strategies may be classified according to their predominant nature as cognitive, mixed, and behavioral, respectively. Each is briefly described below.

Positive reappraisal involves the constructive reinterpretation of a stressful event, emphasizing its potential for personal growth and learning while mitigating its negative emotional impact [34,35]. Social support seeking combines emotional relief with the pursuit of instrumental assistance, and its effectiveness largely depends on the perceived quality and availability of support networks [36,37]. Strategic planning refers to a deliberate, problem-solving approach characterized by the systematic organization of personal resources, a functional evaluation of the situation, and the implementation of a targeted action plan—ultimately enhancing the individual’s sense of perceived control [38,39].

A range of studies has demonstrated significant associations between the use of these active coping strategies and key outcomes, including reduced perceptions of academic stressors, improved psychological well-being, and greater academic self-efficacy [40,41,42]. Moreover, these strategies have been shown to mitigate not only the immediate effects of stress but also its medium- and long-term consequences [43,44], and are associated with lower levels of emotional, cognitive, behavioral, and somatic symptoms [45,46,47].

Despite growing scholarly interest in the dynamics of academic stress, there remains a notable lack of research specifically addressing the mediating role of distinct active coping strategies in the relationship between academic stressors and stress responses. While some studies have proposed mediation models, most rely on broad coping categories, often neglecting the differentiated effects of specific strategies [48,49,50]. In this context, the present study aims to advance understanding of the coping mechanisms involved in the academic stress experience by examining the mediating role of three specific active strategies—positive reappraisal, social support seeking, and strategic planning—in the relationship between perceived academic stressors and self-reported stress responses. This analysis seeks to clarify how these strategies function as protective factors and to inform the design of effective interventions to support the psychological well-being of university students.

**Figure 1:**
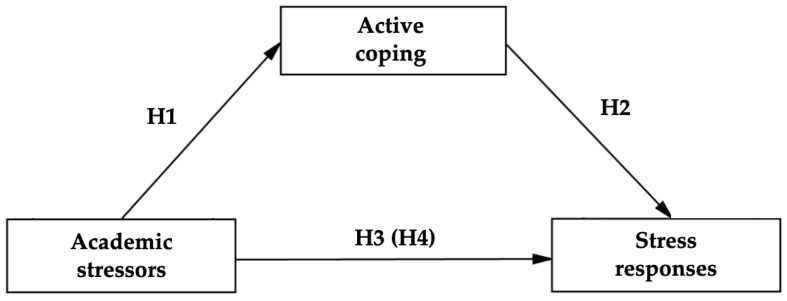
Hypothesized mediation model.

Accordingly, the primary aim of this study is to examine the mediating role of three active coping strategies—positive reappraisal, social support seeking, and strategic planning—in the relationship between academic stressors and self-perceived stress responses. To this end, both the direct and indirect effects of each strategy are analyzed to determine their specific contribution to the academic stress experience. Based on this conceptual framework, the following hypotheses are proposed (Figure 1):

**H1:**  *Academic stressors will exert a negative effect on the use of active coping strategies*.

**H2:**  *Active coping strategies will produce a negative effect on self-perceived stress responses*.

**H3:**  *Academic stressors will exert a direct positive effect on self-perceived stress responses*.

**H4:**  *Active coping strategies will mediate the relationship between academic stressors and stress responses, thereby attenuating the total effect of stressors on self-perceived symptomatology*.

## 2. Methodology

### 2.1. Design and Participants

A descriptive, non-experimental, ex post facto design with a cross-sectional approach was employed [51]. The sample consisted of 1014 undergraduate students from the University of Extremadura, aged between 17 and 63 years (*M* = 20.56, *SD* = 3.50). Participants were selected through cluster sampling, preserving the natural grouping of students within academic cohorts. Regarding gender distribution, 654 participants identified as female (64.5%) and 360 as male (35.5%). In terms of academic year, 28.8% were in their second year *(n* = 292), followed by first-year (27.9%, *n* = 283), fourth-year (23.3%, *n* = 236), and third-year students (20.0%, *n* = 203). As for academic disciplines, the largest group was enrolled in Social and Legal Sciences (43.0%, *n* = 436), followed by Health Sciences (22.6%, *n* = 229), Sciences (17.2%, *n* = 174), Engineering and Architecture (8.8%, *n* = 89), and Arts and Humanities (8.5%, *n* = 86).

### 2.2. Measures

Academic stress was assessed using the three scales comprising the Academic Stress Questionnaire (CEA) [52]:

- Academic Stressors Scale (E-CEA) [53]

This scale measures the frequency with which students perceive academic situations as stress-inducing. It comprises 54 items grouped into eight dimensions: (1) methodological shortcomings of instructors (e.g., “I get nervous or anxious when instructors assign contradictory tasks or activities”); (2) academic overload (e.g., “I get nervous or anxious due to insufficient time to adequately study all subjects”); (3) beliefs about academic performance (e.g., “I get nervous or anxious because I doubt my ability to meet the demands of my program”); (4) public speaking (e.g., “I get nervous or anxious when delivering presentations or speaking publicly for extended periods”); (5) negative social climate (e.g., “I get nervous or anxious due to lack of support from instructors”); (6) perceived lack of content value (e.g., “I get nervous or anxious because what I’m studying seems to have little future utility”); (7) exams (e.g., “I get nervous or anxious as exam dates approach”); (8) participation difficulties (e.g., “I get nervous or anxious because I cannot organize tasks or activities as I would like”). Responses are rated on a 5-point Likert scale (1 = never, 5 = always). Previous studies report high internal consistency, with Cronbach’s alpha coefficients exceeding 0.80 for the full scale [54,55]. In this study, only the total score was used.

- Stress Response Scale (R-CEA) [56]

This scale assesses symptoms of academic stress across three domains: physiological, cognitive, and behavioral. It includes 22 items grouped into five dimensions: (1) physical exhaustion (e.g., “In recent weeks, I feel I have less energy”); (2) sleep disturbances (e.g., “In recent weeks, I sleep restlessly”); (3) irritability (e.g., “In recent weeks, any minor setback irritates me”); (4) negative thinking (e.g., “In recent weeks, I tend to highlight my failures and undervalue my achievements”); and (5) physical agitation (e.g., “In recent weeks, I move excessively without apparent cause”). Items are rated on a 5-point Likert scale (1 = never, 5 = always). Prior research indicates high internal reliability, with Cronbach’s alpha values close to 0.90 [43,54]. Only the total score was analyzed in this study.

- Stress Coping Scale (A-CEA) [33]

This instrument evaluates the coping strategies used by students in response to academic stress. It consists of 23 items distributed across three dimensions: (1) positive reappraisal (e.g., “When facing a problem, I downplay the negatives and emphasize the positives”); (2) social support seeking (e.g., “When dealing with a problem, I ask a trusted family member or friend for advice”); and (3) planning (e.g., “When preparing for exams, I plan my study strategy in detail”). Responses are provided on a 5-point Likert scale (1 = never, 5 = always). The scale shows strong internal consistency, with Cronbach’s alpha values ranging from 0.85 to 0.90 across dimensions [43,57]. Each of the three dimensions was analyzed as a mediating variable in the present model.

Covariates: Sociodemographic variables were collected through brief self-report items embedded in the same online questionnaire. Gender (0 = female, 1 = male), study year (0 = lower years [1st–2nd]; 1 = upper years [3rd–4th]), and academic field (0 = social sciences and humanities; 1 = science, technology, and health) were included as control variables in the analyses.

### 2.3. Procedure

First, a list of faculties from various academic disciplines at the University of Extremadura was compiled. Institutional emails were sent to each faculty to inform them of the study’s objectives, the content of the questionnaire, and the estimated completion time (approximately 25 min). Once institutional approval was obtained, the research team visited classrooms in person to administer the questionnaire collectively, maintaining the natural grouping of each class. Access to the instrument was provided via a QR code, allowing each student to complete it independently through the Google Forms platform during regular class hours. Data were collected between February and April of the 2024–2025 academic year, deliberately avoiding examination periods to minimize potential bias due to evaluative anxiety. The researchers supervised the administration and were available to address any questions raised by participants.

Prior to participation, all students were informed about the study’s aims, the voluntary nature of their involvement, and the exclusive use of the data for scientific purposes. To mitigate social desirability bias, it was emphasized that there were no right or wrong answers. Informed consent was provided digitally before accessing the questionnaire. The instrument was designed with mandatory response fields for all items, preventing submission unless fully completed; consequently, no partial responses were recorded. After data collection, the dataset was carefully reviewed to detect any inconsistencies or out-of-range values. Cases containing evident errors were excluded by listwise deletion, representing less than 1% of the sample. This quality control rendered additional imputation techniques unnecessary.

This study was conducted in accordance with the ethical principles of the Declaration of Helsinki and the Ethical Code of the University of Extremadura.

### 2.4. Data Analysis

The statistical analysis was conducted in two main phases. First, descriptive statistics were computed for all study variables, including arithmetic means, standard deviations, and Cronbach’s alpha coefficients to evaluate the internal consistency of the scales used. In addition, a bivariate correlation matrix was generated using Pearson’s correlation coefficient to examine preliminary associations among academic stressors, coping strategies, and stress responses.

Second, a multiple mediation analysis was carried out using Structural Equation Modeling (SEM) to estimate both the direct and indirect effects of academic stressors on stress symptoms, with active coping strategies included as mediating variables. The model was estimated using AMOS software (Version 26.0), applying the Maximum Likelihood (ML) estimation method together with a bias-corrected bootstrapping procedure (5000 resamples, 95% confidence interval, *p* ≤ 0.05), ensuring robust and precise parameter estimates. An indirect effect was considered statistically significant when the confidence interval did not include zero.

The covariates (gender, study year, and academic field) were included in the SEM model as exogenous predictors, with direct paths specified to the mediators (coping strategies) and the outcome variable (stress responses). This approach controlled for potential confounding effects and ensured the robustness of the main mediation estimates.

Model fit was evaluated using multiple global fit indices [58]: the chi-square test (*χ*^2^; non-significant, *p* > 0.05), the Root Mean Square Error of Approximation (RMSEA < 0.08), the Standardized Root Mean Square Residual (SRMR < 0.06), and both the Comparative Fit Index (CFI) and Tucker–Lewis Index (TLI), with values above 0.95 indicating good model fit.

## 3. Results

### 3.1. Initial Analyses

Prior to conducting the mediation analyses, descriptive statistics, internal consistency estimates, and bivariate correlations among the primary study variables were examined (Table 1). All scales demonstrated satisfactory internal consistency, with Cronbach’s alpha coefficients exceeding 0.80, indicating robust reliability. Descriptive statistics revealed mean scores and standard deviations within a moderate range across all constructs assessed.

Bivariate correlations indicated statistically significant relationships between all variable pairs. Specifically, academic stressors showed a strong positive association with stress-related symptoms (*r* = 0.70, *p* < 0.01). In contrast, academic stressors were inversely associated with the use of active coping strategies, including positive reappraisal (*r* = −0.35, *p* < 0.01), planning (*r* = −0.13, *p* < 0.01), and seeking social support (*r* = −0.10, *p* < 0.01).

Each of these coping strategies was also negatively correlated with stress symptoms, with the strongest association observed for positive reappraisal (*r* = −0.37, *p* < 0.01), followed by planning (*r* = −0.18, *p* < 0.01) and support seeking (*r* = −0.17, *p* < 0.01). Moreover, significant positive intercorrelations were observed among the three coping strategies. Collectively, these preliminary results provide empirical support for the hypothesized mediation framework.

### 3.2. Multiple Mediation Analysis

As indicated in Table 2, with the model adjusted for gender, study year, and field of study, academic stressors exerted a significant and positive direct effect on stress symptoms (*β* = 0.54, *p* < 0.001; 95% CI [0.46, 0.62]). They were also negatively associated with all three active coping strategies—positive reappraisal (*β* = −0.34, *p* < 0.001), planning (*β* = −0.12, *p* < 0.001), and social support seeking (*β* = −0.09, *p* = 0.002)—with the confidence intervals that excluded zero, indicating a lower likelihood of employing these strategies under conditions of elevated academic stress.

Regarding the associations between coping strategies and stress symptoms, both positive reappraisal (*β* = −0.13, *p* < 0.001) and support seeking (*β* = −0.06, *p* = 0.025) were significantly related to lower levels of perceived symptomatology. Planning also exhibited a protective, though more modest, association (*β* = −0.03, *p* = 0.039).

Among the indirect effects, positive reappraisal emerged as the primary mediating mechanism (*β* = 0.09), followed by planning (*β* = 0.02) and support seeking (*β* = 0.01); all were statistically significant, with 95% confidence intervals excluding zero. The total combined indirect effect was also significant (*β* = 0.12, *p* < 0.001; 95% CI [0.08, 0.16]).

The total effect of academic stressors on stress symptoms—encompassing both direct and coping-mediated paths—was substantial and statistically robust (*β* = 0.66, *p* < 0.001; 95% CI [0.59, 0.73]). Given that the direct effect remained significant after accounting for the mediators, the findings are consistent with a partial mediation model.

In relation to the covariates (Table 3), within the same SEM model, identifying as male was associated with greater use of positive reappraisal (*β* = 0.14, *p* = 0.002), lower levels of social support seeking (*β* = −0.11, *p* = 0.021), and fewer reported stress symptoms (*β* = −0.07, *p* = 0.030). Being enrolled in upper study years was linked to higher positive reappraisal (*β* = 0.10, *p* = 0.004) and lower social support seeking (*β* = −0.08, *p* = 0.020). Finally, being in a science, technology, or health field was associated with greater planning (*β* = 0.07, *p* = 0.030).

**Figure 2:**
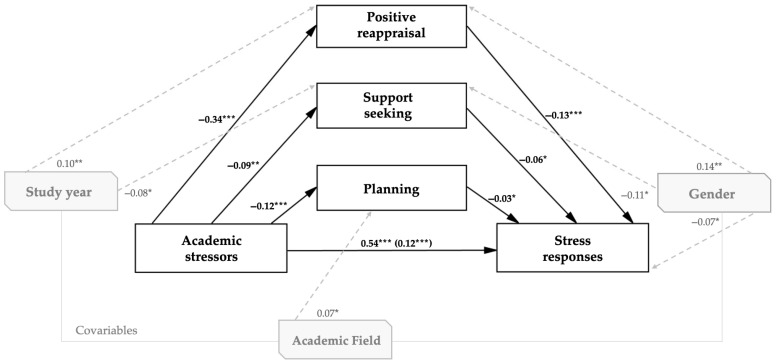
Structural equation model with standardized coefficients. Note. Only significant paths are shown. Solid lines represent the hypothesized paths among core variables, and dashed lines denote significant covariate paths. * *p* < 0.05; ** *p* < 0.01; *** *p* < 0.001.

The SEM showed excellent fit to the data—*χ*^2^ (8) = 7.82, *p* = 0.452; RMSEA = 0.015; SRMR = 0.020; CFI = 0.97; TLI = 0.98—and its structural paths appear in Figure 2. Consistent with the coefficients reported in Table 2, the results support a partial mediation of the coping strategies.

## 4. Discussion

This study examined the mediating role of three active coping strategies—positive reappraisal, strategic planning, and social support seeking—in the relationship between academic stressors and stress-related symptoms among university students, emphasizing both direct and indirect effects within this framework. The results are discussed below in relation to the proposed hypotheses and their consistency with the broader empirical literature.

In line with the first hypothesis, our findings revealed that higher levels of academic stressors were significantly associated with lower engagement in approach-oriented coping strategies. This inverse relationship was strongest for positive reappraisal, followed by planning, and, to a lesser extent, social support seeking. These results offer empirical support for the expected pattern.

Positive reappraisal, a coping strategy that relies on higher-order self-regulatory processes—such as cognitive reframing, inhibition of automatic responses, and emotional modulation—may exhibit diminished effectiveness under conditions of high academic demand. In such contexts, sustained pressure can undermine the availability of executive resources necessary for its proper enactment [59,60,61]. Furthermore, evidence suggests that during episodes of intense academic stress, this strategy may not only become less accessible but can also take on a dysfunctional course, promoting repetitive negative thinking patterns associated with the onset of clinical symptoms in the short term [60].

With regard to planning, although the reduction observed was less pronounced than that associated with positive reappraisal, it remained statistically significant. While planning is a structured, action-oriented coping strategy, its effectiveness depends on executive functions such as temporal organization, decision-making, and consequence anticipation—cognitive capacities that may be impaired under heightened academic pressure [59,62]. This functional decline may decrease students’ propensity to engage in effective planning when they perceive deficits in available time, task clarity, or a sense of control [43,63].

In the case of social support seeking, the observed effect was minimal, suggesting greater resilience to the inhibitory effects of stress. This relative stability may be attributed to the strategy’s comparatively lower cognitive demands and its reliance on external resources—such as peers, family members, or academic mentors—which often remain accessible even when other coping mechanisms are impaired. Nonetheless, previous research has cautioned that social engagement tends to decline among highly stressed students, particularly under adverse contextual conditions, such as social isolation, strained interpersonal relationships, or limited access to support networks [64,65].

These findings support the notion that, as academic demands escalate, students’ capacity to mobilize approach-oriented coping strategies is progressively diminished. This erosion of regulatory capacity increases the likelihood of resorting to more passive, avoidant, or dysregulated responses—particularly in contexts appraised as uncontrollable [32,49,66].

The second hypothesis, which proposed that active coping strategies would negatively predict self-perceived stress responses, was supported by the results. Within the model, positive reappraisal emerged as the most robust protective factor, followed by social support seeking, while planning exerted a comparatively modest influence.

It is noteworthy that positive reappraisal—while identified in the first hypothesis as the coping strategy most vulnerable to academic pressure—also emerged as the most effective in attenuating stress responses. This apparent paradox can be explained by the strategic function that positive reappraisal occupies within the emotional regulation process: it operates early during the cognitive appraisal stage, thereby modulating the impact of the stressor at its source [67,68]. Although its activation is more cognitively demanding under stress, when effectively engaged, its psychological benefits are particularly pronounced [69,70,71]. Moreover, recent research highlights that not only the frequency of use, but also the flexible adaptation of positive reappraisal to the specific demands of each situation, is associated with lower levels of psychological distress—underscoring its importance as a key coping resource in high-pressure academic contexts [72,73].

For social support seeking, the regulatory effect observed was modest yet statistically significant. While the prior literature emphasizes its buffering role in the context of academic stress [74,75], its effectiveness appears contingent upon the nature of its deployment—whether used instrumentally (e.g., to obtain advice or information) or emotionally (e.g., to seek understanding and empathy) [43]. Although generally considered an adaptive strategy, social support seeking may, in certain contexts, foster dependency or avoidance behaviors [76]. Additionally, some studies have reported a mismatch in university populations between the perceived need for support and the actual support received, thereby raising questions about the perceived utility and accessibility of this coping resource [77].

As for planning, it demonstrated a statistically significant negative association with stress symptoms, although its overall effect size was limited. This finding is consistent with previous research suggesting that action-oriented strategies may be effective in contexts that afford some degree of control. However, their regulatory capacity tends to decline when not supported by complementary cognitive or emotional resources [78,79].

It is important to acknowledge that the modest effect sizes observed for planning and social support seeking may, in part, be attributed to the developmental stage of the majority of participants—young adults in the early phases of emerging adulthood [80], many of whom have yet to consolidate a fully mature coping repertoire. As Frydenberg and Lewis [81] point out, coping strategies are shaped by prior learning experiences but require ongoing refinement and adaptation in response to novel and evolving demands, such as those encountered in the university environment.

In this regard, Fisher [82] emphasizes that the transition into university life often elicits a perceived loss of control within a potentially stressful context. This interpretation is reinforced by the findings related to the third hypothesis, which confirmed that academic stressors exert a significant and direct influence on students’ stress responses. Specifically, they contribute substantially to physiological, cognitive, and behavioral symptomatology, thereby consolidating their role as key precipitating factors in the manifestation of stress-related difficulties [54,83]. This result is consistent with prior studies conducted among university populations, which not only corroborate the impact of academic stressors on students’ stress responses, but also highlight their contribution to adaptation challenges and increased levels of subjective distress [19,84].

The final hypothesis posited that active coping strategies would mediate the relationship between academic stressors and stress symptoms. This hypothesis received partial support: positive reappraisal, strategic planning, and social support seeking each emerged as significant mediators, underscoring the modulatory role of cognitive, behavioral, and social resources in navigating high academic demands [48,85,86,87]. However, the persistence of a strong and statistically significant direct effect from stressors to symptoms suggests that, while active coping exerts a buffering influence, it is insufficient on its own to fully offset the impact of perceived academic stress. This finding highlights the critical importance of contextual factors within the academic environment in determining the effectiveness of coping efforts [10].

In this regard, numerous scholars have emphasized the importance of adopting an ecological and context-sensitive perspective on coping—one that integrates not only students’ individual capacities, but also the institutional and relational dimensions that shape their experience and regulation of academic stress [57,88,89]. Accordingly, the present findings reinforce the need for a dual institutional strategy: one that simultaneously promotes the development of students’ coping competencies while also strengthening organizational mechanisms aimed at identifying and addressing stress-inducing elements within the educational system.

In relation to the first strategic axis, it is essential to promote interventions that encourage the flexible application of active coping strategies. Key initiatives include cognitive skills training aimed at the functional reinterpretation of stress, psychoeducational programs, and the integration of mind–body practices (e.g., mindfulness, cognitive-behavioral therapy, yoga); the strengthening of support networks through peer mentoring, tutoring schemes, and community-building initiatives; and foundational training in academic planning and effective study habits, such as time management, active learning techniques, and academic self-efficacy—each representing a priority area for targeted intervention [8,41,90].

Regarding the second strategic axis, there is a need to advance institutional policies that substantially enhance the quality of the academic environment. Recommended measures include the progressive introduction of academic demands over the course of the semester, the adoption of active learning methodologies, the promotion of student involvement in institutional decision-making, and the development of academic planning practices that ensure balanced task distribution, cross-course coordination, and the avoidance of scheduling conflicts [88,91].

Despite the robustness and internal consistency of the findings, this study has several limitations that should be considered when interpreting the results. These are outlined below, alongside suggestions for future lines of research.

First, while the observed effects align with well-established theoretical frameworks—most notably Lazarus and Folkman’s transactional model of stress [26]—the cross-sectional design captures only a static snapshot of the phenomenon. It does not allow for examination of how relationships among stressors, coping strategies, and stress responses unfold over time. Longitudinal studies could offer a more nuanced and dynamic understanding of these processes.

Second, data were collected through self-report measures, a method appropriate for assessing subjective perceptions but susceptible to individual biases, such as social desirability, which may have influenced the accuracy of responses.

Third, coping was assessed through three active strategies that have been widely validated in academic settings [33]. While this approach is consistent with the study’s aims, future research could incorporate a broader range of coping strategies and complementary instruments to enrich and triangulate the findings. Likewise, future studies are encouraged to test alternative SEM configurations that treat academic stressors and stress responses as latent constructs defined by their respective dimensions. This would allow for a more detailed understanding of the unique contribution of each factor and enable more precise identification of specific stress sources, related coping mechanisms, and associated stress responses.

Although the sample was large and diverse, it was drawn from a single institution using convenience sampling, which may limit the generalizability of the findings and introduce potential institutional or cultural biases. Replicating the study in universities with differing profiles—such as virtual, hybrid, or geographically distinct institutions—could strengthen its external validity and expand the applicability of the results.

Finally, despite including sociodemographic covariates (gender, academic year, and academic field) to control for potential confounding effects within the SEM, the study did not conduct multigroup or moderation analyses. Incorporating these approaches in future research would provide a more nuanced understanding of the conditions under which active coping strategies mediate the relationship between academic stressors and stress responses. Such insights could inform the development of more tailored and context-sensitive interventions.

## 5. Conclusions

The findings of this study confirm that a higher level of academic stressors is associated with a lower activation of approach-oriented coping strategies (H1) and that these, in turn, exert a protective effect on self-perceived stress responses (H2). Moreover, the direct impact of stressors on stress-related symptoms is corroborated (H3), as is the partial mediating role of active coping in this relationship (H4). The magnitude of the direct effects indicates that these strategies, although effective, are not sufficient to fully neutralize the impact of perceived stress.

The evidence obtained provides a solid basis for developing more targeted interventions aimed both at strengthening students’ individual active coping skills and at reviewing the institutional conditions that influence the management of academic stress. This supports an integrated approach that acknowledges the dynamic interaction between students and their university environment and promotes more sustainable, healthy, and context-appropriate educational trajectories.

Future research should address limitations such as the cross-sectional nature of the design, the need to broaden the range of coping strategies assessed, and the potential value of conducting multigroup and moderation analyses. These analyses should more systematically incorporate sociodemographic variables—such as gender, academic year, or field of study—whose influence could meaningfully nuance the effects observed.

## Figures and Tables

**Table 1:** Descriptive statistics and correlation matrix.

Variables	*M* (*SD*)	*α*	1.	2.	3.	4.	5.
1. E-CEA	2.70 (0.67)	0.91	1				
2. R-CEA	2.57 (0.74)	0.93	0.70 **	1			
3. PR	2.77 (0.76)	0.85	−0.35 **	−0.37 **	1		
4. PLAN	2.89 (0.80)	0.84	−0.13 **	−0.18 **	0.61 **	1	
5. SS	2.96 (0.97)	0.92	−0.10 **	−0.17 **	0.33 **	0.42 **	1

Note. E-CEA = academic stressors; R-CEA = stress responses; PR = positive reappraisal; PLAN = planning; SS = social support seeking. ** *p* < 0.01.

**Table 2:** Standardized multiple mediation analysis results.

Variables	*β*	Lower CI	Upper CI	*p*-Value
**Direct Effects**				
E-CEA → R-CEA	0.54	0.46	0.62	<0.001
E-CEA → PR	−0.34	−0.39	−0.29	<0.001
E-CEA → SS	−0.09	−0.14	−0.04	0.002
E-CEA → PLAN	−0.12	−0.17	−0.07	<0.001
PR → R-CEA	−0.13	−0.18	−0.08	<0.001
SS → R-CEA	−0.06	−0.10	−0.02	0.025
PLAN → R-CEA	−0.03	−0.05	−0.01	0.039
**Indirect Effects**				
E-CEA → PR → R-CEA	0.09	0.06	0.12	<0.001
E-CEA → SS → R-CEA	0.01	0.00	0.02	0.015
E-CEA → PLAN → R-CEA	0.02	0.01	0.03	0.002
**Total Effect**				
E-CEA → R-CEA	0.66	0.59	0.73	<0.001

Note. E-CEA = academic stressors; R-CEA = stress responses; PR = positive reappraisal; SS = social support seeking; PLAN = planning; *β* = standardized coefficient; CI = 95% Confidence Interval.

**Table 3:** Direct effects of covariates.

Covariates	*β*	Lower CI	Upper CI	*p*-Value
Gender → PR	0.14	0.05	0.23	0.002
Gender → SS	−0.11	−0.19	−0.03	0.021
Gender → PLAN	0.01	−0.06	0.08	0.740
Gender → R-CEA	−0.07	−0.13	−0.01	0.030
Study year → PR	0.10	0.03	0.17	0.004
Study year → SS	−0.08	−0.15	−0.01	0.020
Study year → PLAN	0.02	−0.05	0.09	0.580
Study year → R-CEA	−0.02	−0.06	0.02	0.40
Academic field → PR	0.03	−0.04	0.10	0.420
Academic field → SS	−0.05	−0.12	0.02	0.150
Academic field → PLAN	0.07	0.01	0.13	0.030
Academic field → R-CEA	−0.02	−0.05	0.01	0.40

Note. PR = positive reappraisal; SS = social support seeking; PLAN = planning; R-CEA = stress responses; *β* = standardized coefficient; CI = 95% confidence interval. Gender (0 = female, 1 = male), study year (0 = lower years [1st–2nd]; 1 = upper years [3rd–4th]), and academic field (0 = social sciences and humanities; 1 = science, technology, and health).

## Data Availability

The data are available upon request to the corresponding author.
